# Multiplatform Metabolomics Studies of Human Cancers With NMR and Mass Spectrometry Imaging

**DOI:** 10.3389/fmolb.2022.785232

**Published:** 2022-04-08

**Authors:** Anya B. Zhong, Isabella H. Muti, Stephen J. Eyles, Richard W. Vachet, Kristen N. Sikora, Cedric E. Bobst, David Calligaris, Sylwia A. Stopka, Jeffery N. Agar, Chin-Lee Wu, Mari A. Mino-Kenudson, Nathalie Y. R. Agar, David C. Christiani, Igor A. Kaltashov, Leo L. Cheng

**Affiliations:** ^1^ Massachusetts General Hospital, Harvard Medical School, Boston, MA, United States; ^2^ Department of Biochemistry and Molecular Biology, University of Massachusetts-Amherst, Amherst, MA, United States; ^3^ Department of Radiology, Brigham and Women’s Hospital, Harvard Medical School, Boston, MA, United States; ^4^ Department of Chemistry and Chemical Biology, Northeastern University, Boston, MA, United States; ^5^ Dana-Farber Cancer Institute, Boston, MA, United States; ^6^ Harvard T.H. Chan School of Public Health, Boston, MA, United States

**Keywords:** metabolomics, imaging, mass spectrometry, nuclear magnetic resonance spectroscopy, lung cancer, prostate cancer

## Abstract

The status of metabolomics as a scientific branch has evolved from proof-of-concept to applications in science, particularly in medical research. To comprehensively evaluate disease metabolomics, multiplatform approaches of NMR combining with mass spectrometry (MS) have been investigated and reported. This mixed-methods approach allows for the exploitation of each individual technique’s unique advantages to maximize results. In this article, we present our findings from combined NMR and MS imaging (MSI) analysis of human lung and prostate cancers. We further provide critical discussions of the current status of NMR and MS combined human prostate and lung cancer metabolomics studies to emphasize the enhanced metabolomics ability of the multiplatform approach.

## Introduction

Mass spectrometry (MS) and nuclear magnetic resonance spectroscopy (NMR) are popular analytical techniques used for metabolomics studies. MS allows for the determination of the molecular mass and formula of a compound, but only reveals some structural features. NMR, on the other hand, can be used to reveal the entire structure of a compound. (Bruice, 2016). Both techniques are used to identify and characterize specific compounds, and recently, they have been combined in the rising field of metabolomics: MS is effective in reporting metabolites of extremely low concentrations, while NMR requires only simple sample preparations yet offers highly quantitative results and in vivo-detecting potentials. In addition to providing qualitative information on a single molecule of interest, MS and NMR results can be used to generate metabolic profiles to enable metabolomics investigations of an entire organism. (Nicholson et al., 1999; Nicholson et al., 2002).

Evaluations of quantitative metabolomics changes require techniques that are capable of generating these accurate and sensitive metabolomic profiles. Additionally, to produce robust and statistically significant results, reproducibility is critical. Therefore, not only is there a need for a technique to create comprehensive global metabolic profiles from complex biological samples, but such profiles must also be sensitive and specific. Ideally, such a technique would allow analysis to be performed directly on the samples with minimal or no sample preprocessing to produce high throughput and unbiased results on particular classes of metabolites and compounds. At the same time, the resulting analyses should be highly and equally sensitive to all components and possess a wide dynamic range. (Lenz and Wilson, 2007). Unfortunately, at present, there is no single technique that possesses all the characteristics needed to be considered an ideal global metabolite profiling tool. Thus, use of multiple analytical platforms, such as combining the strengths of NMR with MS, particularly with the recently developed matrix-assisted laser desorption ionization (MALDI) mass spectrometry imaging (MSI) methodologies, for metabolic profiling can maximize understanding and generate more global metabolomics.

In this article, we will first provide a brief background on the methodology of MS and NMR, with particular emphasis on MSI for general readers; followed by presentations of our results on human prostate and lung cancers through collaborative studies of NMR with MSI; and finally, general discussions of the current status of metabolomics studies on these two diseases that utilized both NMR and MS analyses through critical reviews of the current literature.

### Technical Background

#### Mass Spectrometry

MS for metabolomic studies is typically used in combination with chromatography techniques. (Lenz and Wilson, 2007). Two of the most commonly used combinations are gas chromatography-MS (GC-MS) and liquid chromatography-MS (LC-MS). GC-MS has a long history as a major analytical tool in drug metabolism and disposition studies, which goes back over 5 decades. (Tham and Holmstedt, 1965). It has excellent sensitivity and robustness and proves to be an advantage in analytical settings since there exists several extensive databases that can be referenced to identify unknowns. (Lenz and Wilson, 2007). A unique advantage of MS that has been recognized in the early GC-MS studies of drug metabolites is its ability to generate analyte-specific fragment ions, which can dramatically increase the selectivity of the analysis. (Hammar et al., 1968). Furthermore, quantitation can be readily enabled in GC-MS analyses by using isotopically-labeled internal standards, a feature that has been used extensively from the early days of the drug metabolism studies. (Horning et al., 1974). It is, therefore, not surprising that GC-MS enjoys tremendous popularity in the metabolomics arena, as well, since it is one of the most efficient and reproducible analytical platforms in this field. (Beale et al., 2018). However, many classes of compounds such as phospholipids, sugars, nucleosides, and amino acids cannot be directly analyzed using the ion production techniques compatible with GC (electron impact and chemical ionization) due to their polarity and lack of volatility. (Lenz and Wilson, 2007). Therefore, extraction and chemical derivatization must be performed prior to analysis, (Duncan et al., 1971), which can introduce some variability in the process. Additionally, the necessity for precise sample preparation, combined with long run times, yields low throughput, reducing the efficiency of this method.

Derivatization of polar and non-volatile metabolites can be avoided if the analyses are carried out directly in solution using LC-MS; (Dulik et al., 1990); however, it was not until the advent of electrospray ionization (ESI) that the direct analysis of polar and non-volatile metabolites became routine. (Plumb et al., 2002; Idborg-Björkman et al., 2003). Due to its complementarity to GC-MS, LC-MS enjoyed a steady growth in popularity over the past 2 decades in the field of metabolomics and biomarker discovery. Apart from the sample preparation steps common to all metabolomic techniques dealing with *ex vivo* analyses (metabolic quenching, quantitative metabolite extraction and preservation), (Lu et al., 2017), LC-MS usually requires minimal-to-no additional pretreatment/derivatization, which allows for moderate-to-high throughput. It is also extremely sensitive, although it should be noted that it is not equally sensitive to all compounds. Lastly, LC-MS has the capability to perform “contextualized” measurements (*e.g*., detection of metabolites associated with a particular protein that is relevant to a specific pathology). (Pawlowski et al., 2016). As is the case with GC-MS, quantitative metabolomic studies can be readily performed with LC-MS using internal standards produced by stable isotope labeling. (El-Maghrabey et al., 2020). Since the isotopically-labeled species are commercially available only for a handful of common metabolites, a derivatization step is usually required to incorporate differentially labeled functionalities into metabolites in the control sample and in the clinical sample. While introduction of the derivatization step into the workflow obviously decreases the analysis through-put and can introduce bias or noise to the final measurements, a judicious choice of derivatizing reagents frequently allows the detection limits to be improved for a range of metabolites by enhancing their ionization efficiency, fragmentation properties in MS/MS measurements and chromatographic behavior. (Higashi and Ogawa, 2016).

### Mass Spectrometry Imaging

Since MS can only be carried out on tissue extract solutions with homogenized samples, neither GC-MS nor LC-MS can preserve tissue metabolite information, including localizations on tissue pathologies. To investigate spatially localized MS, MSI was developed. The initial implementation of MSI used high-energy projectiles, such as ionized atoms or multi-atomic clusters accelerated to energies up to several tens of keV to dislodge the material from a variety of solid surfaces (including tissue cross-sections) placed under vacuum, a technique which is now known as secondary ion mass spectrometry (SIMS). (Fragu et al., 1994). Since the desorption of the material off the sample surface relies on high-energy ionic beams, which can be focused readily using a range of electrostatic devices, SIMS offers very high spatial resolution (down to sub-μm levels). However, this method of desorption can significantly damage the analyte molecules and very rarely preserves intact metabolites. In the vast majority of cases, the analyses with SIMS imaging are focused on either poly- or indeed mono-atomic fragment ions. In some cases such fragment ions can be used as a means of identifying specific classes of metabolites, (Rodimova et al., 2021), thereby allowing healthy tissues to be differentiated from neoplastic formations. (Gularyan et al., 2020). However, the paucity (or indeed the complete absence) of the molecular ions in the SIMS data sets remains a significant limitation of this technique and perhaps the most significant impediment vis-à-vis its broader utilization in metabolomic studies. While several recent reports indicate that this problem may be solved by employing soft-acting desorption projectiles (such as gas cluster ions beams), (Winograd, 2018), which enables direct detection of intact metabolites with high diagnostic value in cancer tissues, (Tian et al., 2021), this cannot be achieved in SIMS imaging studies carried out with conventional instrumentation.

The problem of excessive ion fragmentation is elegantly resolved by another MSI technique, matrix assisted laser desorption ionization (MALDI), that can produce intact molecular ions from surfaces of tissue slices. (Angel and Caprioli, 2012; Crecelius et al., 2015). The tissue damage is avoided by coating the imaged surface with a uniform thin layer of the MALDI matrix, a weak organic acid that has high absorptivity at the wavelength of the laser used to irradiate the surface during the measurements. The latter feature allows the laser energy to be channeled very selectively to the matrix molecules, which allows any damage to the endogenous biomolecules to be minimized or indeed completely avoided during the desorption process. The acidic nature of the matrix molecules allows them to serve as a reservoir of protons, thereby facilitating analyte ionization. (Norris and Caprioli, 2013; Buchberger et al., 2018). Although the spatial resolution offered by MALDI MSI (10–100 μ m per pixel) is relatively modest compared to SIMS imaging, it can readily generate molecular ions for a wide range of biomolecules with little-to-no fragmentation, and map small molecules, metabolites, lipids, and small proteins onto tissue histology features at single-cell scale. (Gilmore et al., 2019). Until recently, all MSI analyses were carried out exclusively *ex vivo* on the removed specimens, but incorporation of the so-called ambient ionization methods (such as the desorption/electrospray ionization, or DESI) (Takáts et al., 2004) in the workflow may allow at least some analyses to be carried out in the intraoperative format. (Brown et al., 2021; King et al., 2021). However, it must be stressed that even though these analyses are carried out *in vivo*, they cannot be accomplished without surgical intervention to provide access to the internal tissues.

### Nuclear Magnetic Resonance

Unlike MS, NMR metabolomics analysis is simpler and more straightforward, and fast developments in the medical utilizations of MRI technologies further offer a potential of *in vivo* metabolomics observations. NMR has been used to investigate biofluid compositions dating back to the 1980s (Gadian, 1982; Nicholson et al., 1984; Iles et al., 1985; Bell et al., 1989; Gartland et al., 1989; Nicholson et al., 1989). The technique provides detailed structural information of organic molecules and enables a large number of compounds to be identified and catalogued. Additionally, NMR allows for the monitoring of changes in metabolic profiles following different treatment stages or natural disease progression through the fluctuation of detected metabolite concentrations. (Gadian, 1982; Nicholson et al., 1984; Iles et al., 1985; Bell et al., 1989; Gartland et al., 1989; Nicholson et al., 1989). The technique fulfills many of the criteria that enable it to be a powerful analytical tool for metabolomic investigations. For instance, it requires minimal-to-no sample preparation, is cost-effective, unbiased, rapid, robust, reproducible, quantitative, nonselective, and nondestructive. Particularly, following the discovery of high resolution magic angle spinning (HRMAS), (Cheng et al., 1996; Cheng et al., 1997), intact biological tissues now can be measured directly without sample preparation and with the capability of preserving tissue pathological structures. (Cheng et al., 2000; Vandergrift et al., 2018; Berker et al., 2019). Therefore, now NMR has been used to investigate all types of biological samples from biofluids, tissue extracts, to intact tissues. However, it should also be pointed out that comparing with MS, NMR is less sensitive when measuring molecules of low concentrations below micro- or nano-moles.

Considering the compensatory strengths and weaknesses of these modalities, their multiplatform usage would present results at a more elevated level than any of them alone could reach. The benefits of a combined analysis method have been demonstrated through multiple studies that have used both MS and NMR techniques to isolate, identify, and characterize compounds that could potentially serve as therapeutic agents, including for lung and prostate cancer. These studies analyzed the behavior of human cancer cell lines when treated with various compounds suspected to have anti-tumor properties. (Zhang et al., 1995; Sundaram et al., 2009; Dias et al., 2018; Gordon et al., 2018; Chen et al., 2019; Balusamy et al., 2020; Dyshlovoy et al., 2020; Gao et al., 2020; Dhaneesha et al., 2021). Furthermore, MS and NMR have been used together in clinical studies investigating lung cancer and prostate cancer detection and characterization. (Hao et al., 2016; Clendinen et al., 2019; Dudka et al., 2020; Lima et al., 2020).

Below, we will use our experimental examples to specifically demonstrate the value of combining HRMAS NMR with MALDI MSI in studies of human lung and prostate cancer metabolomics.

## Results

Development of HRMAS NMR for intact tissue analysis has led to studies of various human malignancies, including brain, breast, prostate, lung, rectal, thyroid cancers, etc., which can be seen in a systematic review with more than 200 references covering the period 1997–2017 can be found in literature. (Dietz et al., 2017). While HRMAS method allows us to measure disease metabolomics and preserve tissue architectures for evaluations with quantitative pathology after NMR analyses and requires a very small tissue size of less than 10 mg, such a volume is still ‘macroscopic’ and contains pathological mixtures considered by histology. Given the known pathological heterogeneity of human diseases, it is extremely important to understand the pathological cellular origins of these observed metabolites and their distributions among disease pathologies at the cellular levels that can be probed by MSI. Recently, of note, MSI studies on human tissue specimens, as well as samples generated from human cancerous cells, have been reported for prostate, (Banerjee et al., 2017; Kurreck et al., 2018; Wang et al., 2017; Morse et al., 2019; Mutuku et al., 2019; Randall et al., 2019), and lung cancers, (Marko-Varga et al., 2011; Végvári et al., 2013; Li et al., 2015; Tsubata et al., 2017; Bensussan et al., 2020), respectively.

### Human Prostate Cancer and Polyamines

Statistical data on the natural history of prostate cancer (PrCa) show that >70% of patients diagnosed by PSA screening will likely experience indolent disease with little impact on well-being. For about 17% of newly PSA-diagnosed patients, however, aggressive PrCa proliferation ensues, truncating life expectancy. (Siegel et al., 20152015). At present, no reliable clinical test can differentiate between these two groups. Polyamines (PA) are essential elements for optimum growth of cells. (Cohen, 1998; Gerner and Meyskens, 2004; Wallace, 2009). PA concentrations, particularly that of spermine are increased by 110–240% in cancer compared to normal tissues for most human malignancies (brain, colon, kidney, oral, breast, heart, blood, etc.). (Fair et al., 1972; Dunzendorfer and Russell, 1978; Matsuda et al., 1978; Dimery et al., 1987; Lamster et al., 1987; Becciolini et al., 1991; Ernestus et al., 1996; Cañizares et al., 1999; Ernestus et al., 2001; Weiss et al., 2002; Ekegren and Gomes-Trolin, 2005; Magnes et al., 2014; Meana et al., 2016). However, PrCa is an exception, with spermine in cancer tissues reported at only about 60% of that seen in benign tissue. (Dunzendorfer and Russell, 1978; Cipolla et al., 1990; Cipolla et al., 1996; Fu et al., 1998; Giskeødegård et al., 2013; Shukla-Dave et al., 2016; Braadland et al., 2017; Sandsmark et al., 2017). This relationship likely reflects spermine’s exceptionally high concentrations in prostate (millimolar level, mM), which is at least 5–10 times higher than in tissues from all the other measured organs, and are measurable with ^1^H MRSI. (Basharat et al., 2014). In addition to prostate tissue, spermine concentrations in the prostatic fluid have been reported as high as ∼59 ± 45 mM. (Serkova et al., 2008; Serkova et al., 2009). Such unusually high concentrations in prostate suggest spermine to be an endogenous inhibitor that may slow PrCa growth in prostate, with spermine’s microenvironment being toxic to PrCa cell growth in prostate. (Smith et al., 1995).

Using HRMAS ^1^HNMR followed by quantitative histology, (Cheng et al., 1996; Cheng et al., 1997), we previously showed statistically significant correlations between concentrations of spermine and the amount of histologically-benign epithelial (Hb Epi) prostatic cells and glands in human cancerous prostates. (Cheng et al., 2001). However, as previously discussed, HRMAS NMR alone cannot prove that spermine was indeed generated or resided in the Hb Epi cells.

**FIGURE 1:**
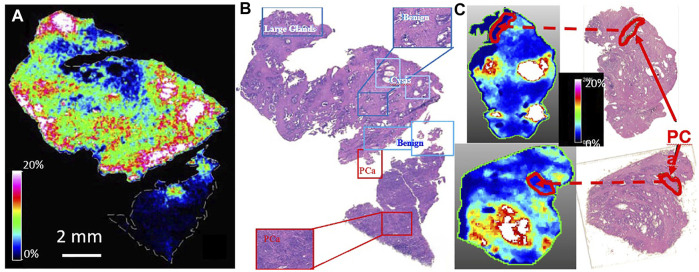
MALDI MSI Confirmed HRMAS 1H NMR prediction of associations of spermine with histologically benign epithelia. De-identified human prostate tissues from PCa patients were evaluate by histology and measured with MALDI MSI. **(A)**. Map of 12C-Spermine (m/z: 203.223 ± 0.001Da) **(B)**. Histology identifications of prostate tissue pathologies including, benign glands, cysts, and PCa lesions that located spermine onto the benign epithelial glands. **(C)**. Verifications of spermine locations with other cases.

However, using MALDI MSI, (Kurreck et al., 2018), we were able to locate ^12^C-spermine (m/z: 203.223 ± 0.001Da) onto Hb Epi, as shown in Figure 1, while spermine on the PrCa lesions appeared to be below the detection limit. From these maps, for the first time, we could visualize and confirm the differential localizations of spermine in prostates. In this figure, the color white indicated that spermine intensity was above the 20% threshold defined for the plots; the lowest spermine intensities (dark blue) were seen in the histology identified PrCa glands (circled in black and linked in red dashed lines between MSI and histology images).

This proof of spermine relationship to prostate pathologies is of critical importance because of its proposed PrCa inhibitory effects. (Smith et al., 1995). Considering spermine’s proposed inhibitory characteristics during prostate cancer development and progression, results investigating spermine biosynthetic activities in human prostates have been reported. (Kaul et al., 2010). Results from this study shown that the expression levels for mRNA of spermine anabolic and catabolic enzymes: ornithinedecarboxylase (ODC1), S-adenosylmethionine decarboxylase (AMD1), and antizyme (OAZ1) vary in different cellular components in tissue, and correlate with patient PSA velocities, (Kaul et al., 2010), that may explain the immune-like responses of prostate healthy epithelia in biosynthesis of spermine for inhibitions of cancer growth while located in the prostate. The current confirmative observation of localized spermine distributions in prostate with MALDI MSI supports further studies that are critical in differentiating aggressive from indolent PCa for disease evaluations and patient personalized treatment strategies.

### Human Lung Cancer and Metabolomic Distributions

Despite decreasing incidence in both men and (more recently) women, lung cancer (LuCa) still kills nearly 160,000 annually in the United States alone; it is responsible for more deaths than breast, colon and prostate cancers combined, (National Cancer Institute Surveilance, 1975; Siegel et al., 2020), mostly due to the often late detections of the disease. Currently, low-dose spiral CT (LDCT) is considered the most sensitive imaging tool for detecting small and early-stage LuCa lesions. (Oudkerk et al., 2020). However, logistical and scientific concerns, ranging from the costs to potential radiation hazard, limit LDCT’s use as a screening tool for general populations. (Oudkerk et al., 2020; Toumazis et al., 2020). Therefore, developments of a simple, non- or minimally-invasive screening test that can alert suspicious signs of early malignancy are needed to triage patients to advanced technologies, such as LDCT, for LuCa early detections.

To search for such LuCa screening metabolomics biomarkers, we used HRMAS NMR and analyzed 93 pairs of human LuCa tissue and serum samples and 29 healthy human sera. A number of potential metabolite candidates capable of differentiating LuCa characteristics were identified, including glutamate, lipids, alanine, glycerylphosphorylcholine, glutamine, phosphorylcholine, etc. (Berker et al., 2019).

**FIGURE 2:**
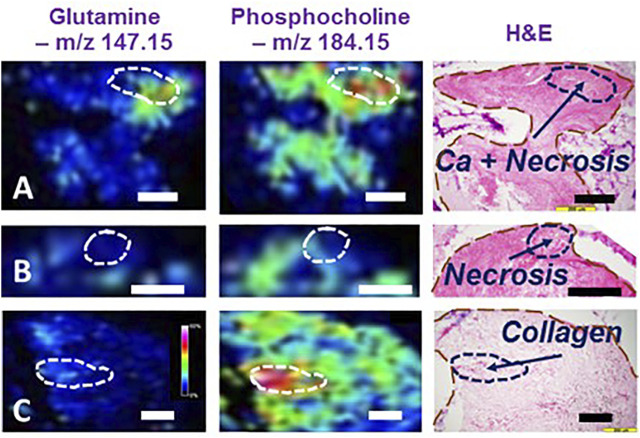
Illustrations of MALDI MSI metabolomic maps of human lung cancer tissues. Interpretation of MALDI MSI results obtained from three human lung cancer cases according to tissue histology. **(A)** SCC, Stage I, M, 72.2y.o. **(B)** Adeno, I, F, 71.8, and **(C)** SCC, I, F, 59.6. In **(A)**, the MSI region with high glutamine and phosphorylcholine intensities was correlated with a histological region featured by cancer lesions mixed with necroses. However, observing necroses alone, as shown in **(B)**, neither of these two metabolites were overexpressed in the MSI. Furthermore, interpretation of the presence of phosphorylcholine requires caution for its increase in collagen was seen in **(C)** *Black and white bars denote 200 μm.*

We investigated these HRMAS NMR indicated metabolites on LuCa tissues with MALDI MSI, using tissue samples from a group of six LuCa patients (SCC = 3, 2F, 1M, Age = 75.2 ± 4.5; Adeno = 2, 2F, 1M, Age = 66.3 ± 6.3). Our resulting MALDI MSI, shown in Figure 2, illustrates that LuCa and necrosis identified by histology in case A presented increased metabolic intensities for both glutamine and phosphorylcholine, findings not seen for necrosis alone (B). Of note, interpretation of phosphorylcholine increase requires caution, as its increase in collagen can also be observed in (C). However, in that case, the increase in glutamine would be nearly absent. *This observation emphasizes the importance of conducting metabolomic evaluations, rather than merely observing individual metabolites*.

**FIGURE 3:**
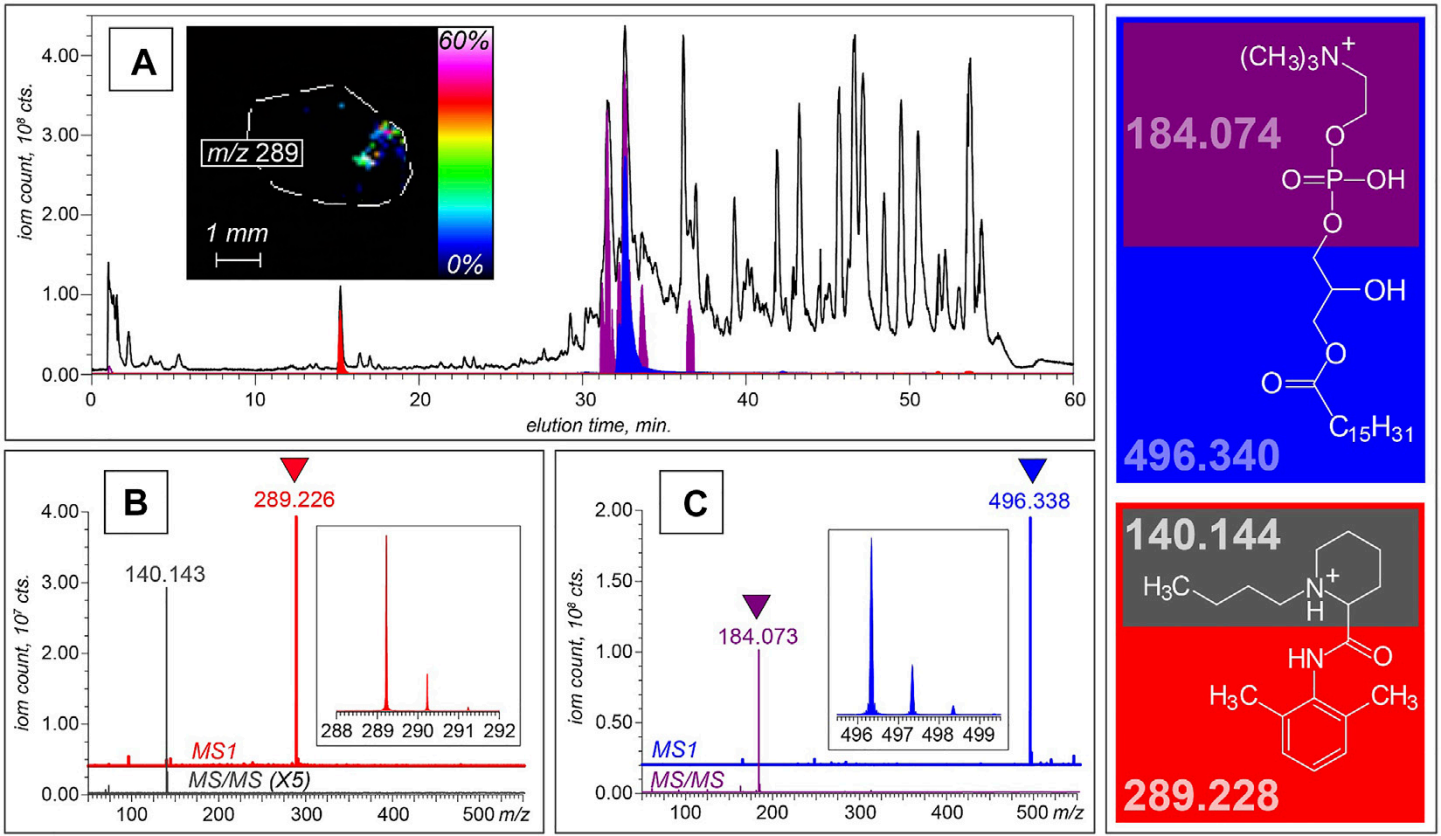
LC-MS and LC-MS/MS analyses of non-volatile components of serum collected from a lung cancer patient. **(A)** XICs of intact molecular ions at m/z 496.3 (blue) and m/z 289.2 (red), and a fragment ion at m/z 184.1 (purple) plotted on the background of the total ion chromatogram (shown with a black trace). The MS and MS/MS datasets shown at the bottom of this panel were collected at elution time of 15 min **(B)** and 32 min **(C)**, allowing these two species to be identified as bupivacaine and LPC16:0, respectively. The structures of both molecular ions and the most abundant fragments alongside their calculated masses are shown in the right-hand-side diagram. The inset in panel **A** shows a spatial distribution of the bupivacaine signal within the cross-section of a biopsy tissue obtained with MALDI MSI.

The HRMAS NMR proposed metabolomics list can be further expanded by analyzing sera from cancer patients and control healthy subjects using LC-MS, which can offer a dramatic increase in sensitivity compared to HRMAS NMR and, therefore, may be better suited for the further biomarker discovery. This advantage is illustrated in Figure 3, which shows the results of metabolite profiling in a lung cancer patient’s serum using reversed-phase LC-MS. The single LC-MS measurement reveals the presence of over a hundred unique species in the 100–3,000 Da mass range. The high resolving power of the MS (a Fourier transform ion cyclotron resonance MS in this particular case) allows the mass measurements for the lower molecular weight species (<600 Da) to be carried out with accuracy that is sufficient for establishing their empirical formulae.

In addition to acquiring high-resolution mass data, the high data acquisition rate allows the fragment ion mass spectra (the so-called tandem mass spectra, or MS/MS) to be generated for the most abundant ionic species in each chromatographic peak. This feature allows specific classes of tumor-attenuated metabolites to be identified based on the presence of unique structurally diagnostic fragment ions in MS/MS spectra. For example, lysophosphatidylcholine (LPC) molecules are known to be actively degraded in solid tumors, where they are used as a material for generating fatty acids that are incorporated into the membranes of malignant cells. (Raynor et al., 2015). This results in a dramatic attenuation of LPC levels in neoplastic formations, (Raynor et al., 2015), and several previous studies demonstrated association of several LPCs with tumors and their aggressiveness, including lung, (Guo et al., 2012; Han et al., 2021), ovarian, (Kim et al., 2014), prostate, (Goto et al., 2015), colorectal, (Li et al., 2013), and laryngeal (Yu and Wang, 2021) cancers. The fatty acid components of LPCs display a range of structures, which differ from each other by the chain length, the hydroxylation status and the presence of unsaturated bonds, giving rise to a plethora of molecular ions with unique masses in LC-MS datasets. However, gas-phase fragmentation of these ions almost invariably results in facile dissociation of the phosphocholine head group (C_5_NO_4_PH_15_^+^), allowing these fragment ions (*m/z* 184.07389) to be used as markers of phosphocholine-containing phospholipids (both LPCs and sphingomyelin). (Brugger et al., 1997). The extracted ion chromatogram for this fragment ion (shown in purple in Figure 3A) displays eight peaks within the 31–37 min elution window, and each LPC species in this series can be readily identified based on the accurate mass data. This is illustrated in Figure 3C, where the molecular ion giving rise to the diagnostic fragment C_5_NO_4_PH_15_^+^ at elution time of 33 min has *m/z* value of 496.338. The high resolving power of the MS allows this value to be measured with confidence exceeding 5 ppm, a mass accuracy sufficient to identify this species as LPC (16:0/0:0), or 1-palmitoylphosphatidylcholine. In this particular case, the accurate mass measurement afforded by the high resolving power of the instrument alone is sufficient to establish the metabolite identity. However, this task can be accomplished by lower-resolution instruments, as well, as long as they allow MS/MS measurements to be carried out: indeed, the 496-to-184 transition is unique to 1-palmitoylphosphatidylcholine, and therefore can be used to identify this LPC with high confidence. Furthermore, metabolite identification tasks are greatly facilitated by using publicly available databases, such as the Human Metabolome database and Metlin. (Wishart et al., 2013; Guijas et al., 2018). Of caution, when analyzing spectra at the high m/z range (>1,000), potential contributions from different ditopologies of the same compound can be substantial that should be considered to avoid overestimations of the number of unique metabolites. (Mahieu and Patti, 2017).

Although metabolomic studies are primarily focused on endogenous species, LC-MS also provides a means of detecting xenobiotics in clinical samples. This allows a wealth of information to be obtained on metabolism and disposition of a range of therapeutic agents without the need to design and carry out any additional measurements. For example, a distinct chromatographic peak at 15 min in the LC-MS dataset discussed earlier reveals the presence of an anesthetic bupivacaine, which was identified on the basis of both accurate mass measurement (within 7 ppm) and MS/MS data (a unique fragment at *m/z* 140.143), as shown in Figure 3B. Detection of serum bupivacaine points at the use of this local anesthetic during the biopsy collection, but of course LC-MS alone cannot establish its introduction route. The latter task can be uniquely accomplished using MALDI MSI (inset in Figure 3A).

## Discussion

Using a multiplatform approach including both MS and NMR techniques allows complementary information to be obtained and provides a more holistic and comprehensive understanding of the underlying mechanisms and compounds responsible for observed metabolomics alterations due to physiological and pathological processes. Additional collaborative studies involving HRMAS NMR and MALDI MSI can improve disease metabolomics at the intact tissue level, as demonstrated by the observations presented here. While these MSI observations clearly advance metabolomics knowledge on these human diseases evaluated with NMR, such combined studies have, unfortunately, not been reported in literature.

At present, NMR and MS related reports on human diseases are usually conducted in one of two directions: they are either used to isolate and elucidate characteristics of a suspected active compound; or combine the collected data to conduct metabolic profile analyses. The focus of these studies is centered on constructing more efficient and targeted treatment strategies for these diseases. Studies of combined use of both NMR and MS methods seen in literature will be critically discussed below, while other studies with single use of either NMR or MR are beyond the scope of our discussion.

### Human Prostate Cancer

Results from a human urine study confirmed the compatibility of using NMR and MS analysis techniques together to provide a deeper understanding of PrCa development. (Lima et al., 2020). The design of the study included analyzing metabolic profiles of individuals with and without PrCa and reported significant alterations in the concentrations of 21 metabolites. Eight of these metabolites associated with PrCa were previously reported by other metabolomic studies, however, the other 13 were reported for the first time through the NMR and MS combined study. Additionally, 14 metabolic pathways were found to be significantly altered and dysregulated, most of which were associated with energy metabolism, with the most significantly altered pathway being associated with amino acid metabolism. (Lima et al., 2020). Using both NMR and MS to analyze the samples helped ensure the robustness of the data, and the study results revealed that alterations in amino acids and energetic metabolism play a critical role in the development and progression of PCa. (Lima et al., 2020).

Combined NMR and MS analyses have also been used to differentiate between disease subgroups. PrCa subtypes were investigated through analyses of metabolic alterations related to disease development and progression. Biomarkers between benign and malignant samples were analyzed using both NMR and LC-MS, with focuses on metabolic profiles that could identify the aggressiveness and progression of disease according to Gleason scores reflecting tumor aggressiveness and gene fusion, *TMPRSS2-ERG,* of progression. (Dudka et al., 2020). Results showed that increases in hypoxanthine and arginine were correlated with tumors of high Gleason score, and associated with PrCa occurrence and progression. Additionally, increases in β-oxidation and purine metabolism often reported for PrCa can be mainly attributed to *TMPRSS2-ERG*-negative tumors. The altered metabolites measured in the study provide molecular information on the underlying biochemical mechanisms of PrCa and can guide therapeutic targets. In fact, these results led to the consideration that ERG-positive and ERG-negative PrCa might be somewhat different diseases and require different treatment strategies. (Dudka et al., 2020).

Metabolic profiles have also been used to predict treatment efficacy. Analyses of nontargeted and fused MS and NMR data from preoperative metabolic alterations associated with PrCa have assisted a better understanding of its biochemical recurrence (BCR), (Clendinen et al., 2019), defined as increases in patient blood prostate specific antigen (PSA) levels following radical prostatectomies. The study was conducted with human blood serum samples using fused LC-MS and NMR data sets to discover metabolic fingerprints associated with BCR. (Clendinen et al., 2019). Their results showed that patients who remain in remission exhibit higher glucose levels and lower concentrations of lactic acid. However, patients who experienced BCR exhibited lower glucose levels and higher concentrations of lactic acid, as well as significantly altered lipid profiles. Additionally, correlation and pathway mapping analysis revealed that metabolites involved in amino acid metabolism displayed significant relative differences between remission and BCR patients. (Clendinen et al., 2019). While further research is needed to identify the source behind the altered metabolites, the reported results allowed for preoperative BCR prediction, which could help avoid unnecessary surgery and associated complications in patients for whom a radical prostatectomy may not be an adequate solution.

### Human Lung Cancer

The MS and NMR multiplatform approach has also been used to discover potential LuCa prognostic biomarkers that may predict patient responses to treatments. Serum metabolic profiles from 25 LuCa patients undergoing chemotherapy and/or radiation were studied, and metabolites as temporal biomarkers of clinical outcomes were analyzed. (Hao et al., 2016). Characterizations of serum metabolic profiles from 134 samples from these 25 patients prior to, during, and following their standard chemotherapy and/or radiation suggested that data acquired using GC-MS could reflect progression and survival status, whereas the data acquired from NMR associated better with cancer stage and type. Specifically, GC-MS data revealed that tridecan-1-ol, octadecan-1-ol, and hydroxylamine were abundant in patients of poor survival rates, while glutamine, proline, valine, threonine, and tyramine were abundant in patient groups that had better chance of survival. Additionally, relatively higher concentrations of hydroxylamine were found in the group of patients who were noted to progress with disease despite therapy, and glucopyranose and theronic acid were metabolites found at relatively higher levels in patients that showed little signs of disease progression. On the other hand, the NMR-derived metabolic profiles indicated stage and type as reflected in the serum profiles, with eight metabolites facilitating the discrimination of cancer staging between stages 1 and 2, versus stage 3. (Hao et al., 2016). A further LuCa subgroup analysis shown that 19 differentially abundant spectral features were identified as being able to discriminate non-small cell lung cancer patients into squamous and adenocarcinoma subtypes. These findings suggest that there is variability in metabolic profiles of LuCa patients associated with staging, prognosis, and survival. (Hao et al., 2016).

## Conclusion

At present, without the existence of a single technique that can create quantitative, robust, and sensitive metabolomic profiles, multiplatform approaches have been employed to maximize results. In this article, we presented our findings from NMR and MSI multiplatform human prostate and lung cancer studies. Our results suggest that combined NMR and MSI metabolic analyses of tissue samples from cancer patients can be used to interpret NMR observed cancer metabolomics with MSI localizations onto disease pathologies. Our presented NMR and MSI results, as well as our discussions on multiplatform NMR and MS studies on these two diseases seen in literature, demonstrate the benefits of a multidiscipline metabolomics approach to diagnosing and categorizing human prostate and lung cancers, that cannot be achieved by evaluations using either modality alone.

## Methods

### Literature Review

The multiplatform studies reviewed here were identified through advanced PubMed article searches. The articles were first evaluated on their relevance to the topic of a multiplatform analytical approach to investigate topics surrounding and pertaining to prostate and lung cancers. After reading the study in its entirety, the relevance was reevaluated based on its primary focus and aim. Those that were included had experimental designs aimed to provide new insights into the diseases through the investigation of nonspecific groups and populations and used novel approaches or reported original findings. The date of publication was also taken into consideration, with all but two cited articles being published within the last decade.

### HRMAS ^1^HNMR.

Detailed experimental procedures of HRMAS NMR measurements for human prostate cancer tissues (Vandergrift et al., 2018) and human lung cancer tissues and sera (Berker et al., 2019) can be found in the cited references.

### MALDI-MSI for Human Prostate Cancer Specimens.

Prostate samples were cryosectioned to 10 µm thickness, and thaw-mounted onto indium-tin-oxide (ITO) slides and serial sections were obtained for hematoxylin and eosin (H&E) staining. Using an image stitching program, whole tissue images were obtained (Zeiss Observer Z.1, Oberkochen, Germany) using a plan-apochromat lens (20 ×). α-Cyano-4-hydroxycinnamic acid (5 mg/ml) matrix was dissolved in 70:30 methanol: water with 0.1 TFA and sprayed using a TM-sprayer (HTX imaging, Carrboro, NC) onto the prostate tissues. A two-pass spray protocol with parameters consisted of a flow rate (0.17 ml/min), spray nozzle velocity (1,200 mm/min), spray nozzle temperature (75 °C), nitrogen gas pressure (10 psi), track spacing (2 mm) was used.

A 9.4 T SolariX XR FT-ICR MS (Bruker Daltonics, Billerica, MA) operating in positive ion mode was used for the mass spectrometry imaging experiments. Prior to the MSI run the instrument was mass calibrated over the *m/z* range of 154–3,000 using tune mix solution (Agilent Technologies, Santa Clara, CA). The MSI parameters included the laser repetition rate set to 1,000 Hz, the step size was set to 50 μm, and each pixel consisted of 200 laser shots. SCiLS Lab software (version 2020c premium, Bruker Daltonics, Billerica, MA) was used to visual ion images. Ion images and mass spectra were viewed and processed using SCiLS Lab software (version 2019c premium, Bruker Daltonics, Billerica, MA), in which the dataset was normalized to the total ion current (TIC). Compound annotations were putatively assigned using accurate mass of Δppm <2.5 and cross matched using the Metlin metabolite database.

### MALDI-MSI for Human Lung Cancer Specimens.

The lung cancer tissues were sectioned at MGH to a thickness of 12 µm and placed on indium tin oxide coated glass slides. Slides were then coated with matrix (2,5-dihydroxybenzoic acid) using a Bruker ImagePrep sprayer device using standard methods. MALDI-MSI experiments were performed on a Bruker ultrafleXtreme MALDI-TOF/TOF mass spectrometer using a lateral resolution of 50 µm (50 laser shots/pixel, laser power 60%, shot frequency 2 kHz, acquisition range 0–3,000 m/z). Compounds in the MALDI-MSI images were identified using either MS/MS and/or accurate m/z measurements after internal standard alignments after acquisition of the mass spectra.

### Metabolomics LC-MS

Polar metabolites were extracted from 10 µl plasma by adding 90 µl acetonitrile:methanol 75:25 with 0.1% formic acid. Samples were vortexed for 1 min and then placed on ice for 5 min. Samples were centrifuged at 14,400 *x* g for 10 min at 4°C and supernatant was transferred to an autosampler vial and analyzed immediately. LCMS data were collected on a solariX 7T FT-ICR instrument (Bruker, Billerica, MA) externally calibrated using sodium formate clusters, coupled to an Agilent 1100 HPLC system. Samples (5 µl) were resolved using a Kinetix C18 (2.6 µm) 50 × 2.1 mm analytical column (Phenomenex, Torrance, CA) with mobile phases A. 0.1% formic acid and B. acetonitrile with 0.1% formic acid running the following gradient at 0.2 ml/min: 0 min 2%B, 5 min 2%B, 45 min 80%B, 50 min 80%B, 51 min 2% B, 60 min 2%B. The mass spectrometer was operated in positive mode with the capillary voltage set to 4500 V, dry gas flow and temperature of 8 L/min and 200 °C. The data size of 128kword was collected from 50–1,000 m/z.

## Data Availability

The raw data supporting the conclusion of this article will be made available by the authors, without undue reservation.

## Supplementary Material

Table S1 Comparison of HRMAS NMR and MALDI MSI Techniques
