# Supplementary figures and images for: Multiplatform Metabolomics Studies of Human Cancers With NMR and Mass Spectrometry Imaging
Source: Front Mol Biosci. 2022 Apr 8;9:785232. doi: 10.3389/fmolb.2022.785232 (PMC9024335; doi:10.3389/fmolb.2022.785232)

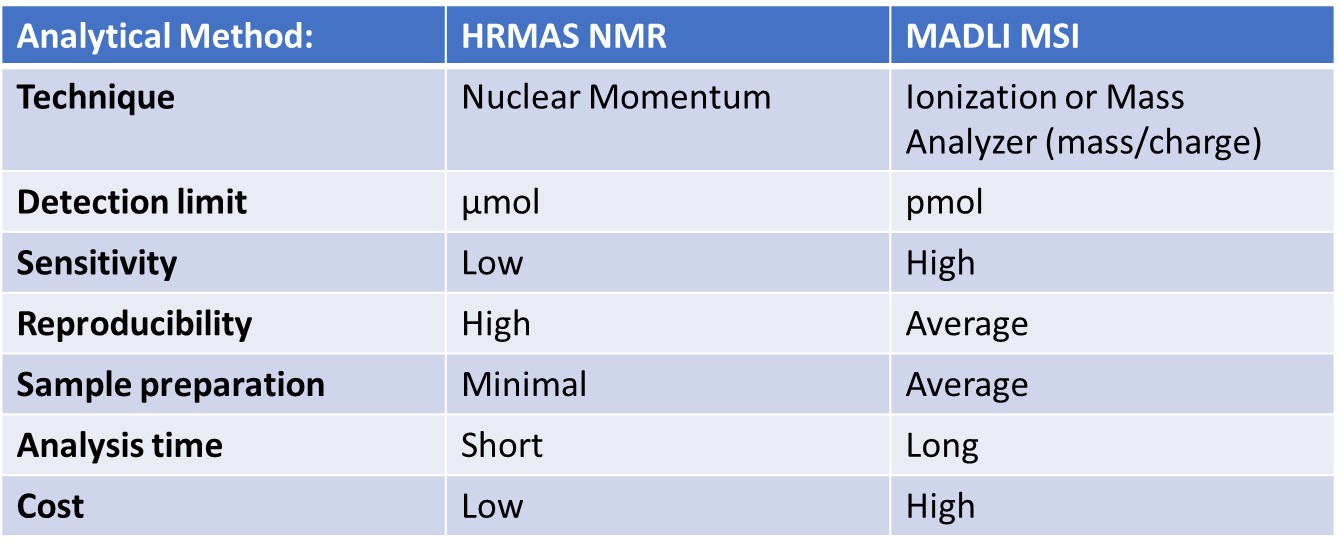

Supplement: Supplementary file 1 [file Image1.JPEG]
